# Mating Attempts and Sustained Interest Behaviors of Wild Boars ( *Sus scrofa* ) Toward a Dead Conspecific

**DOI:** 10.1002/ece3.73848

**Published:** 2026-06-16

**Authors:** Akino Inagaki, Kei Okuda, Maximilian L. Allen, Shinsuke Koike

**Affiliations:** ^1^ Wildlife Management Center Tokyo University of Agriculture and Technology Tokyo Japan; ^2^ Hiroshima Shudo University Hiroshima Japan; ^3^ Illinois Natural History Survey, Prairie Research Institute University of Illinois Champaign Illinois USA; ^4^ Institute of Global Innovation Research Tokyo University of Agriculture and Technology Tokyo Japan

**Keywords:** Davian behavior, interaction, mating, necrophilia, thanatology

## Abstract

Responses of animals to dead conspecifics are rarely characterized from ecological or comparative thanatological perspectives. Here, we report the behavioral responses of three wild boars ( *Sus scrofa* ) toward the carcass of an adult female conspecific, which was monitored using camera traps throughout the entire decomposition process. These observations were made in Nikko City, Japan, between October 1 and November 11, 2023. One adult male exhibited mounting behavior toward the carcass, providing evidence of necrophilic behavior. This behavior is rarely documented in mammals; according to our literature review, this is the 11th case in mammals and the 4th in terrestrial mammals. Furthermore, the adult male's interest in the carcass persisted for approximately 17 days after the first visit, continuing after mounting events and including behaviors such as stepping on the carcass and resting near the carcass. Documentations of necrophilic behavior are often based on single observations, which may mean that important elements of comparative thanatology, such as behavioral shifts and responses associated with different carcass decomposition stages, are being overlooked. None of the individuals consumed soft tissues, suggesting that visits to the carcass were not motivated by feeding on carrion. These observations highlight sustained interest in a conspecific carcass without feeding and reveal pronounced individual variation in behavioral responses. We suggest that monitoring carcasses can provide valuable insights into comparative thanatology beyond traditional carrion ecology.

## Graphical abstract

We documented behavioral responses of wild boars ( *Sus scrofa* ) to a conspecific carcass using camera traps throughout the full decomposition process. One adult male exhibited necrophilic behavior and sustained non‐feeding interactions with the carcass, highlighting pronounced individual variation. These findings suggest that carcass monitoring can provide valuable insights into comparative thanatology beyond traditional carrion ecology.

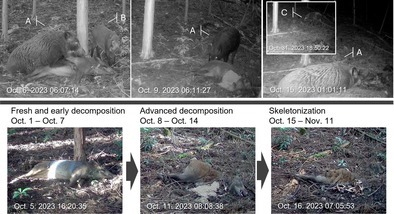

## 1 Introduction

Animals die from two main causes: being killed (e.g., victims of accidents, enemies, and predators), and dying from natural causes (e.g., sickness and old age) (Forbes and Carter 2015). Carcasses resulting from predation are often avoided by conspecifics, likely due to the proximity of the predator (Moleón and Sánchez‐Zapata 2021). Carcasses from natural death are typically consumed rapidly by scavengers and decomposers immediately after death (DeVault et al. 2003; Newsome et al. 2021; Inagaki et al. 2025). Consequently, responses of conspecific to death from an ecological perspective is rarely documented and characterized.

On the other hand, comparative thanatology studies how animals respond behaviorally and psychologically to dead individuals can provide insights into a species' life history traits and behavioral and evolutionary theory (Anderson 2016; Gonçalves and Biro 2018). In vertebrates, several responses toward dead conspecifics have been reported, including avoidance, feeding, transport, and complex responses—such as carrying and alloparental care of dead infants, and necrophilic behaviors (Reggente et al. 2016; Gonçalves and Biro 2018; Bercovitch 2020; Toyoda et al. 2024). However, observations of complex responses in mammals are limited to case reports in non‐human primates, proboscids, and cetaceans—taxa characterized by social behavior, extensive alloparental care, and relatively large brains. Therefore, the responses of conspecifics to death have many gaps or biases in information, both in ecology and thanatology.

We report distinct behaviors of three wild boars ( *Sus scrofa* ) in responses to an adult female conspecific carcass during the complete decomposition process. Wild boars are among the most widely distributed ungulate species in the world (Massei and Genov 2004) and exhibit a dynamic social structure across space and time (McIlraith et al. 2025). Although they are omnivorous and primarily consume plant material (Ballari and Barrios‐García 2014), wild boars are also known to scavenge carrion (Inagaki et al. 2020) as well as rarely cannibalism (Cukor et al. 2020). In contrast, the response of wild boars to a dead conspecific remains unknown when viewed from the perspective of comparative thanatology.

### 1.1 Field Observations

**FIGURE 1:**
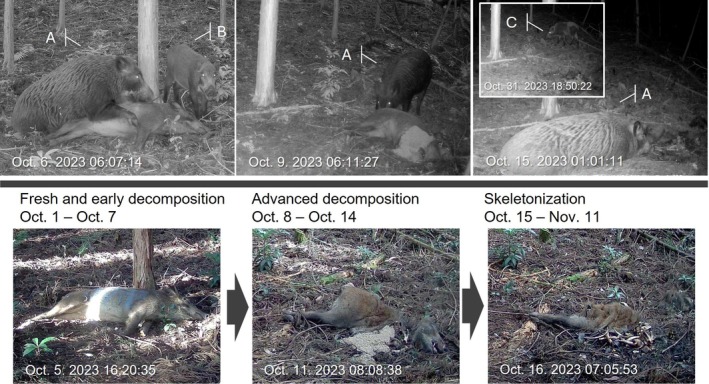
Visits by three wild boars (individuals A, B, and C; top row of photos) to a dead conspecific at different decomposition stages (bottom row of photos). The dates and times of photographs extracted from videos are indicated in the figures. These were recorded using camera traps in Nikko City, Tochigi Prefecture, Japan between October 1 and November 11, 2023.

We deployed a wild boar carcass (an adult female not lactating, an estimated body mass 50 kg) in a *Cryptomeria japonica* plantation and placed a camera trap (HCLS4G, Hyke Inc., Japan) to monitor activity at the carcass in Nikko City, Tochigi Prefecture, Japan (36.786101° N, 139.712321° E) between October 1 and November 11, 2023, as part of a study aimed at identifying vertebrate scavengers of wild boar carcasses. The carcass was obtained from a culling effort (captured by a snare trap) for prevent agricultural damage, and capturing methods were most minimizes pain and distress in accordance with the “Welfare and Management of Animals Act” (Ministry of the Environment) and “Specified Wildlife Conservation and Management Plan” (Tochigi Prefecture). We handled the carcass according to the guidelines of the American Society of Mammalogists (Sikes and Animal Care and Use Committee of the American Society of Mammalogists 2016) and the guidelines for animal research set forth by the Mammalogical Society of Japan (2009), and IACUC approval at the Tokyo University of Agriculture and Technology was not required. The camera was programmed to record 30 s video clips upon motion detection with no delay between triggers and to capture one interval recording per day to document carcass decomposition stages (i.e., fresh, early decomposition, advanced decomposition, and skeletonization; Probst et al. 2020; Figure 1).

In total, we obtained 335 videos (167.5 min), of which 189 videos (94.5 min; 56.4%) recorded wild boars. Other vertebrates recorded include raccoon dogs ( *Nyctereutes procyonoides* ), masked palm civets ( *Paguma larvata* ), sika deer ( *Cervus nippon* ), jungle crows ( *Corvus macrorhynchos* ), and White's thrush ( *Zoothera aurea* ). Vertebrate scavenging was confirmed only for raccoon dogs (total feeding time = 88 s) and jungle crows (total feeding time = 15 s) from the fresh to advanced decomposition stage, but the carcass was primarily consumed by invertebrates.

We defined videos of wild boars as belonging to the same detection event unless the interval between consecutive (previous) videos was ≥ 30 min, in which case they were considered a new detection event. During the observation period, we recorded 15 detection events involving three wild boars (individuals A, B, and C; Table 1; Figure 1). Individuals were identified from video data by three independent observers based on morphological characteristics, including body size, body shape, external genitalia, and snout length. From October 6 to October 18, individual A (an adult male) visited the carcass 12 times (179 videos), including four visits during early decomposition, six during advanced decomposition, and two during skeletonization. In three of these visits, individual B (a juvenile female) accompanied individual A (62 videos). Individual B was recorded alone only once (one video during advanced decomposition) and showed no apparent carcass‐directed behavior. In addition, individual C (a subadult of unknown sex) visited the site twice during the skeletonization stage (October 31 and November 6; nine videos), engaging only in exploratory movement around the carcass without direct interaction. Based on these observations, only individual A exhibited clear carcass‐directed interest behavior. We therefore quantified 15 behavioral categories of individual A at 1‐s intervals (see ethogram in Table 2) and calculated the proportion of time allocated to each behavior within each detection event.

**TABLE 1:** Summary of 15 detection events by three wild boars (Individuals A, B, and C) to a dead conspecific at different decomposition stages in Nikko City, Tochigi Prefecture, Japan.

Event	Decomposition stage	Duration	Individual
1	Early decomposition	Oct.6 6:02:50–Oct.6 6:28:22	A, B
2	Early decomposition	Oct.6 18:34:55–Oct.6 18:59:36	A
3	Early decomposition	Oct.7 4:21:01–Oct.7 4:49:54	A
4	Early decomposition	Oct.7 5:26:48–Oct.7 6:06:00	A
5	Advanced decomposition	Oct.8 18:30:25–Oct.8 18:33:25	A
6	Advanced decomposition	Oct.9 6:10:41–Oct.9 6:12:27	A
7	Advanced decomposition	Oct.9 19:23:15–Oct.9 19:24:18	A
8	Advanced decomposition	Oct.10 5:26:59–Oct.10 5:48:02	A
9	Advanced decomposition	Oct.12 20:18:16–Oct.12 20:18:46	B
10	Advanced decomposition	Oct.13 16:21:13–Oct.13 16:21:43	A
11	Advanced decomposition	Oct.14 23:56:13–Oct.15 1:20:15	A, B
12	Skeletonization	Oct.15 9:14:56–Oct.15 9:15:26	A
13	Skeletonization	Oct.18 16:28:02–Oct.18 16:30:12	A, B
14	Skeletonization	Oct.31 18:50:09–Oct.31 18:53:29	C
15	Skeletonization	Nov.6 0:54:23–Nov.6 0:57:12	C

**TABLE 2:** Behavioral categories and their definitions recorded at 1‐s intervals during carcass‐directed interest behavior of a wild boar (individual A). These observations were recorded from October 6 to October 18 in Nikko City, Tochigi Prefecture, Japan.

Behavior	Description
Mounting	Bending the forelegs and straddling a carcass, often accompanied by tail raising
Nosing	Touching or gently pushing a carcass with the snout
Rolling	Pushing and turning a carcass using the snout
Chin‐resting	Placing the chin on a carcass while remaining still
Nibbling	Lightly biting at a carcass with the front teeth
Feet‐moving	Stepping on the carcass with shifting pressure from the feet
Resting	Lying on the ground with the body relaxed and immobile
Pausing	Stopping all movement and standing still for a brief period
Sniffing	Bringing the snout close to a carcass to investigate its scent
Flehmen	Lifting the upper lip with the mouth slightly open, often accompanied by a brief pause
Yawning	Opening the mouth widely and slowly, often accompanied by a deep inhalation
Digging	Displacing soil or ground debris using the snout or the legs
Treading	Stepping repeatedly around a carcass in a confined area
Others	Behaviors that did not fit any predefined category but were clearly observed and distinct from all other classified actions
Unknown	Behavioral state could not be identified due to obstruction, poor visibility, or unclear posture

### 1.2 Literature Review

Following the observation of mounting behavior in individual A (see Section 2), we searched the peer‐reviewed documentation of necrophilic behavior in mammals. We conducted a literature search on January 17, 2025 and March 18, 2026 using Web of Science with the terms “necrophili*,” “necrocoit*,” or “Davian behavior.” We excluded books, non‐English publications, reports describing only hypotheses, and reports describing sexual arousal toward carcasses without copulatory behavior (i.e., copulation or attempted copulation). Of the 68 articles retrieved, five reported necrophilic behavior in non‐human mammals. We conducted iterative snowball sampling by searching the Literature Cited sections of the papers that we reviewed (e.g., Nettles et al. 2026), which resulted in five additional articles that were not uncovered in the original search. From these 10 studies, we compiled information on the passive (dead) species and sex, active male species, carcass observation method, carcass observation period, and number of necrophilic behavior (Table 3).

**TABLE 3:** Peer‐reviewed literature documenting necrophilic behavior in mammals. The first five articles were obtained through a literature search on Web of Science, and the remaining five articles were obtained through a snowball sampling.

Articles	Species			Carcass observation		
	Passive (dead) species	Sex (dead)	Active male species	Method	Period	Number of necrophilic behavior
Colombo and Mori (2020)	Eurasian badger ( *Meles meles* )	♀	Eurasian badger ( *Meles meles* )	Camera trap	12 days	1 time
Methion and Díaz López (2021)	Short‐beaked common dolphin ( *Delphinus delphis* )	♀	Common bottlenose dolphin ( *Tursiops truncatus* )	Direct observation	1 day	1 time
Kincaid et al. (2022)	Common bottlenose dolphin ( *Tursiops truncatus* )	♀	Common bottlenose dolphin ( *Tursiops truncatus* )	Direct observation	1 day	1 time
Toyoda et al. (2024)	Stump‐tailed macaque ( *Macaca arctoides* )	♀	Stump‐tailed macaque ( *Macaca arctoides* )	Camera trap, Direct observation	3 days	9 times
Grandi et al. (2025)	South American sea lion ( *Otaria flavescens* )	♀	South American sea lion ( *Otaria flavescens* )	Direct observation	3 days	Unknown (multiple times)
Dickerman (1960)	Thirteen‐lined ground squirrel (*Ictidomys tridecimlineatus*)	♂	Thirteen‐lined ground squirrel (*Ictidomys tridecimlineatus*)	Direct observation	1 day	1 time
Brown (1962)	Short‐finned pilot whale ( *Globicephala macrorhynchus* )	♀	Short‐finned pilot whale ( *Globicephala macrorhynchus* )	Direct observation	1 day	1 time
Wilson (1979)	New Zealand fur seal ( *Arctocephalus forsteri* )	♀	New Zealand sea lion ( *Phocarctos hookeri* )	Direct observation	1 day	1 time
Harris et al. (2010)	Harbor seal ( *Phoca vitulina* )	♀	Sea otter ( *Enhydra lutris* )	Direct observation	1 day	1 time
Best et al. (1981)	Fur seal (*Arctocephalus pusillus*)	♀	Elephant seal ( *Mirounga leonina* )	Direct observation	Unknown (nearly 46 days)	33 times

## 2 Results and Discussion

**FIGURE 2:**
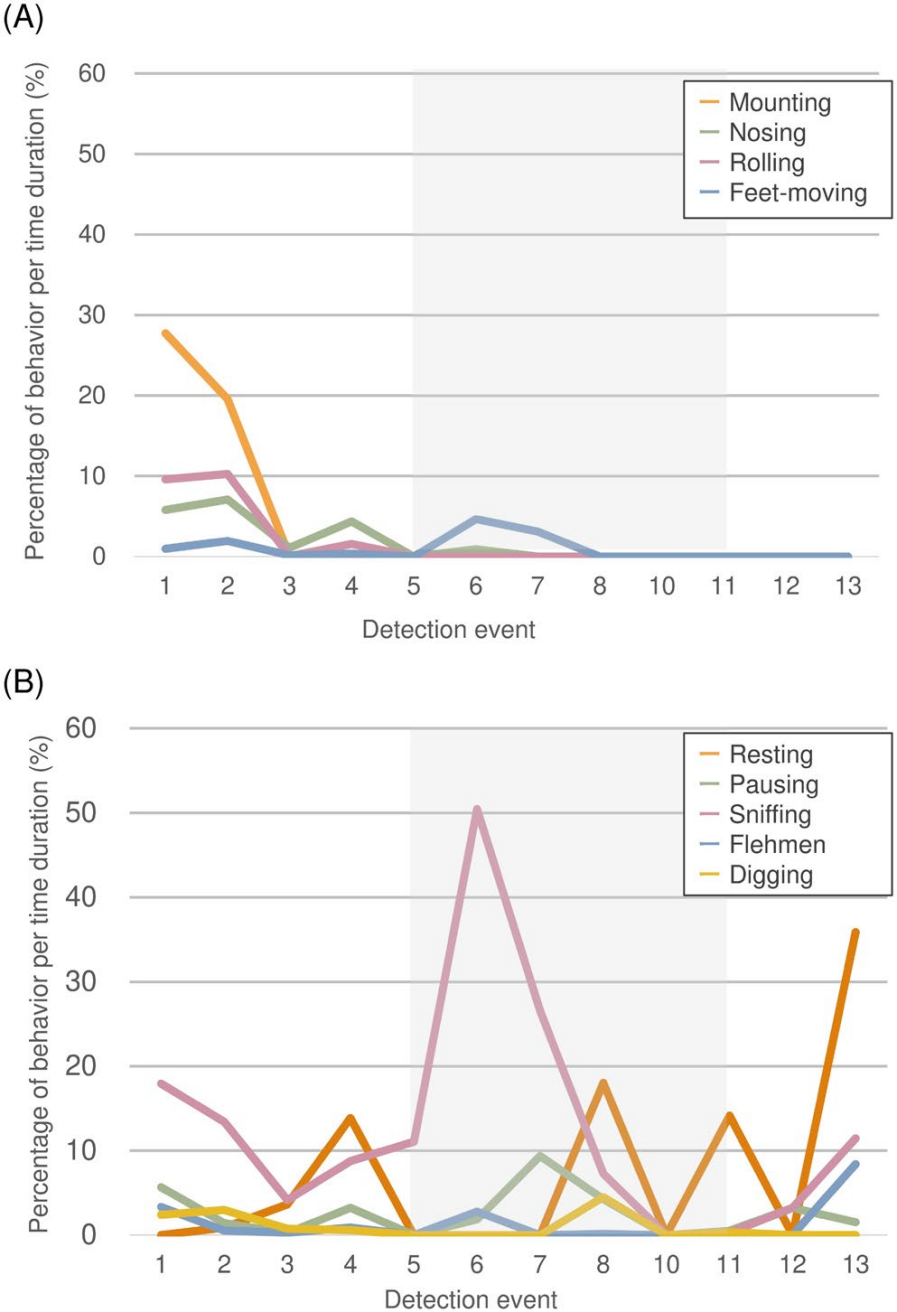
Percentage of total time allocated to each behavior by individual A during each event. (A) Behaviors involving contact with the carcass. (B) Behaviors not involving contact with the carcass. The gray bands indicate different carcass decomposition stage (i.e., advanced decomposition). Behaviors categorized as “chin‐resting,” “nibbling,” “yawning,” and “treading” accounted for less than 4% of the behaviors recorded for each detection event. No characteristic behaviors were identified from the “other” or “unknown” categories. These categories were excluded from the calculation. These were recorded using camera traps in Nikko City, Tochigi Prefecture, Japan between October 6 and October 18, 2023.

Among atypical behaviors exhibited by individual A, mounting was recorded for a total of 719 s (12.0 min) during events 1 and 2 (Figure 2, Video 1). In addition, nosing—which is considered a minor component of wild boar mating behavior but an important element of courtship in domestic pigs (Eguchi et al. 1999)—was frequently observed during event 1 (5.81%) and event 2 (7.09%) and decreased as carcass decomposition progressed (e.g., event 3: 1.10%, event 4: 4.38%, Figure 2, Video 1). Chin‐resting—another behavior associated with mating in wild boars (Eguchi et al. 1999)—was also observed during events 2 and 4. However, it accounted for only a small proportion of the overall behavioral budget, and we did not observe the typical behavioral sequence from chin‐resting to mounting (Eguchi et al. 1999). Together, these observations provided evidence for necrophilic behavior, defined here as attempted copulation directed toward a dead conspecific or closely related species.

**VIDEO 1:** Mounting and nosing behaviors of wild boar individual A (adult male) toward a dead conspecific (adult female). This video was recorded at 06:06 on October 6, 2023 in Nikko City, Tochigi Prefecture, Japan. Video content can be viewed at https://onlinelibrary.wiley.com/doi/10.1002/ece3.73848.

Necrophilic behavior has been rarely reported worldwide because our literature review identified 10 articles of necrophilic behavior in mammals (Colombo and Mori 2020; Methion and Díaz López 2021; Kincaid et al. 2022; Toyoda et al. 2024; Grandi et al. 2025; Dickerman 1960; Brown 1962; Wilson 1979; Harris et al. 2010; Best et al. 1981; Table 3). Three records were reported in terrestrial mammals, although this apparent rarity may partly reflect observational bias. In mammals and birds, necrophilia has been suggested to arise from interactions between reproductive hormonal changes and passive stimuli derived from carcass posture or appearance (Lehner 1988; Tomita and Iwami 2016; Toyoda et al. 2024), or as a strategy by socially disadvantaged males to secure mating opportunities (Colombo and Mori 2020). In this study, individual A was not of a body size indicative of a socially disadvantaged status. Moreover, the carcass was positioned laterally rather than in a prone posture and was frequently rolled by individual A during events 1 and 2, making it unlikely that carcass posture alone elicited mounting behavior. While mounting was observed when the carcass remained relatively fresh, nibbling—considered an investigative behavior (Signoret et al. 1975) was recorded during events 1, 2, and 4 (0.78%–1.89%). These observations suggest that, in addition to the visual condition of the carcass, individual A may have detected some form of chemical cue, subsequently engaging in investigative behaviors that culminated in mounting. One potential proximate factor is the estrous state of the deceased female. However, because no information was available on her reproductive condition at death other than that she was not lactating, this possibility cannot be evaluated in the present study. In fact, during early decomposition (events 1–4), sniffing was more frequently observed in events 1 and 2 when mounting occurred than in events 3 and 4, supporting the hypothesis proposed by Eguchi et al. (1999) that olfactory investigation plays a key role in wild boar courtship behavior. However, because individual A first visited the carcass 4.6 days after placement, it remains unclear whether hormonally relevant chemical signals persisted. Consequently, we were unable to obtain conclusive evidence regarding the proximate cues that led individual A to exhibit necrophilic behavior.

Evidence of necrophilic behavior in mammals has been documented primarily through single, opportunistic observations (Table 3). Only two cases of extended carcass observations have been reported among mammals (i.e., terrestrial mammals: Colombo and Mori (2020), marine mammals: Best et al. (1981), Table 3). Brief records of necrophilic behavior may overlook important elements in comparative thanatology, such as behavioral shifts and responses associated with different carcass decomposition stage. In this study, although the total monitoring period spanned 42 days, the interval between the first and last visits by individual A extended for approximately 17 days, with mounting observed during two early visit events on 6 October.

Notably, behaviors indicative of sustained interest toward the carcass continued after the occurrence of mounting. Specifically, among behaviors involving direct contact with the carcass, feet‐moving increased during the advanced decomposition stage (Figure 2; events 6 and 7). This behavior may represent tactile assessment of carcass condition; however, feet‐moving directed toward carcasses or other specific objects has rarely been documented as a characteristic behavior in wild boars (Erdtmann and Keuling 2020; Cukor et al. 2020), highlighting the need for further observations. Among behaviors without direct contact, sniffing occurred more frequently during advanced decomposition than during skeletonization (Figure 2; advanced decomposition: 7.28%–50.47%, skeletonization stage: 0.28%–11.45%). In addition, resting increased from early decomposition to skeletonization (Figure 2, Video 2). Sniffing is considered a behavior for gathering information about the carcass and its surroundings, thus the co‐occurrence of sniffing and resting within the same visit events suggests that individual A did not appear to exhibit behavioral avoidance toward the carcass regardless of its decomposition stage.

**VIDEO 2:** Wild boar individual A (adult male) resting beside a dead conspecific (adult female). This video was recorded at 01:02 on October 15, 2023 in Nikko City, Tochigi Prefecture, Japan. Video content can be viewed at https://onlinelibrary.wiley.com/doi/10.1002/ece3.73848.

An important aspect of these observations is that, although wild boars made physical contact with the conspecific carcass, such contact was unlikely to be motivated by feeding. Indeed, none of the recorded individuals exhibited scavenging behavior, defined here as the consumption of carcass tissues. This finding is consistent with previous reports (Probst et al. 2017; Leivers et al. 2023). Although cannibalism in wild boars has been reported by Cukor et al. (2020), it has been suggested that such behavior does not represent a common or habitual feeding strategy. However, individual A was recorded chewing rib bones of the carcass during the skeletonization stage (Video 3), and similar observations have been reported by Probst et al. (2017). However, our observations did not allow for inference about the functional significance of bone chewing. Because wild boars are monogastric, calcium acquisition from bones may be more feasible than in ruminants. Nevertheless, the lack of soft tissue consumption suggests that nutritional deficiency is an unlikely primary explanation for this behavior. Instead, bone chewing may reflect a different functional context, potentially as part of a broader suite of sustained interest behaviors directed toward the carcass. Collectively, these results highlight that wild boar in this study maintained sustained interest in a conspecific carcass for purposes other than direct feeding. These sustained interests and repeated physical contact to conspecific carcasses may increase the risk of transmitting diseases such as African and classical swine fever, even in the absence of scavenging behavior. These findings also raise an important question: could complex, comparative thanatological responses to a dead conspecific be observed in more diverse taxa?

**VIDEO 3:** Wild boar individual A (adult male) chewing a rib bone of a dead conspecific (adult female). This video was recorded at 16:28 on October 18, 2023 in Nikko City, Tochigi Prefecture, Japan. Video content can be viewed at https://onlinelibrary.wiley.com/doi/10.1002/ece3.73848.

## 3 Conclusion

Our observations suggest that the study of carrion ecology can yield important insights not only into scavenger‐related processes, but also into comparative thanatology. Although wild boars are social animals, their social organization is considerably less complex than that of primates or cetaceans. In addition, wild boars have high reproductive capacity and do not engage in prolonged or intensive parental care. Although no information was available on the pre‐mortem relationships between the observed individuals and the deceased female, behavioral responses to the carcass clearly differed among individuals A, B, and C. In particular, the occurrence of necrophilic behavior and sustained interest from individual A highlights pronounced individual‐level variation. On the other hand, this study is based on a single case, and it remains unclear whether the various conspecific responses to a wild boar carcass—such as necrophilic behavior, resting beside the carcass, or bone chewing—have any functional or adaptive significance, or instead reflect phylogenetic constraints. Addressing these questions will require the accumulation of further observations and comparative analyses across cases and taxa. In this context, the rapidly increasing use of camera traps to monitor carcasses (Newsome et al. 2021) provides a valuable opportunity to evaluate conspecific responses and to advance comparative thanatological research.

## Author Contributions

**Akino Inagaki:** conceptualization (lead), data curation (equal), formal analysis (lead), funding acquisition (lead), investigation (lead), resources (lead), visualization (lead), writing – original draft (lead), writing – review and editing (lead). **Kei Okuda:** data curation (equal), visualization (supporting), writing – review and editing (supporting). **Maximilian L. Allen:** conceptualization (supporting), visualization (supporting), writing – review and editing (supporting). **Shinsuke Koike:** funding acquisition (supporting), visualization (supporting), writing – review and editing (supporting).

## Funding

This study was financially supported partly by Japan Society for the Promotion of Science KAKENHI grants (no. JP24K23917). A.I. and S.K. were also supported by the Institute of Global Innovation Research and University Research Administration Center in Tokyo University of Agriculture and Technology.

## Conflicts of Interest

The authors declare no conflicts of interest.

## Data Availability

All recorded observations are provided within the manuscript.
